# Subclinical Hearing Deficits in Noise-Exposed Firefighters

**DOI:** 10.3390/ijerph191711028

**Published:** 2022-09-03

**Authors:** Hillary A. Snapp, Natasha Schaefer Solle, Barbara Millet, Suhrud M. Rajguru

**Affiliations:** 1 Department of Otolaryngology, University of Miami Miller School of Medicine, Miami, FL 33136, USA or; 2 Department of Medicine, University of Miami Miller School of Medicine, Miami, FL 33136, USA; 3 Department of Interactive Media, University of Miami, Coral Gables, FL 33146, USA; 4 Department of Biomedical Engineering, University of Miami, Coral Gables, FL 33146, USA; 5 RestorEar Devices LLC, Kirkland, WA 98033, USA

**Keywords:** noise-induced hearing loss, age-related hearing loss, occupational hearing loss, surveillance, firefighters, early detection, subclinical

## Abstract

Noise-induced hearing loss (NIHL) is the most prevalent occupational disease in the world and firefighters are at increased risk of NIHL due to their frequent exposure to hazardous levels of noise during service. Adverse effects of NIHL include acceleration of age-related hearing loss and an increased risk of cognitive decline. A critical challenge in addressing NIHL is the delayed clinical presentation of symptoms and lack of sensitive tools for early detection. To study the early clinical symptoms of NIHL in this high-risk group, we collected hearing function data including behavioral audiometric thresholds and distortion product otoacoustic emissions (DPOAEs) in 176 firefighters during annual physical assessments. Results revealed significant deficits in cochlear outer hair cell function in the presence of normal audiograms. Additionally, 55% of firefighters self-reported changes in hearing, while 20% self-reported concerns about their balance. This study is the first to characterize DPOAEs in firefighters who display decreased DPOAE amplitudes with increasing years in the fire service. These effects were observed even when controlling for hearing loss and age and are suggestive of a link between hearing loss and occupational exposure to hazardous noise.

## 1. Introduction

Noise exposure is the leading cause of acquired hearing loss, a significant global health burden affecting nearly 500 million people worldwide [1]. Overexposure to hazardous levels of noise leads to the early onset of inner ear structural changes [2,3,4,5], accelerating age-related hearing loss [2,6,7,8]. Noise alone is estimated to account for 30% of hearing loss, making exposure to noise the leading preventable cause of hearing loss [9]. Hearing loss is highly prevalent in aging adults and is associated with a number of chronic health conditions [10,11,12,13], most notably cognitive impairment [14,15,16].

Noise-induced hearing loss (NIHL) is the most prevalent occupational disease in the world, with an estimated 1.3 billion people suffering from hearing loss due to noise overexposure [17]. Noise exposure often results in a perceptible temporary shift in hearing thresholds that may be associated with other auditory symptoms such as tinnitus or muffled hearing. In temporary threshold changes induced by hazardous noise, hearing thresholds typically recover [18]. However, animal studies on the interaction of age and noise exposure demonstrate that hearing loss is exacerbated in noise-exposed populations and permanent threshold shifts in hearing are delayed. Additionally, the loss of cellular structures in the inner ear, in particular the cochlear outer hair cells (OHCs), is exacerbated in aged, noise-exposed populations compared to aged populations without noise exposure [6]. These findings demonstrate that early noise exposure renders the inner ears significantly more vulnerable to aging.

Otoacoustic emissions can be used to monitor early changes in the micromechanical function of the cochlear OHCs arising from noise exposure [19,20,21,22]. Distortion product otoacoustic emissions (DPOAEs) provide frequency-specific information about OHC function and are reduced or absent in cases presenting with OHC damage [23]. Changes in the amplitude of the DPOAE response can occur in the absence of hearing loss, which has been suggested to be a sensitive marker of preclinical inner ear damage [5,19,24,25,26].

Noise overexposure can also damage the vestibular periphery (see summary by Stewart et al. [27]). Preclinical work based on different animal models has demonstrated that, depending upon the noise characteristics, noise overexposure often leads to cellular damage in the peripheral vestibular system with a well-characterized effect on the response of otolith organs [28,29,30,31]. Studies using vestibular evoked myogenic potentials (VEMPs) [31,32,33,34] have provided evidence of the same in individuals with NIHL and in military veterans. Latency shifts, reductions in VEMP amplitudes, increased VEMP thresholds, and absent VEMPs have been shown in NIHL populations with abnormalities correlated to both hearing thresholds and history of noise exposure.

Occupational groups such as firefighters are at particularly high risk of noise-induced changes to the inner ear due to chronic exposure to hazardous noise levels [35,36,37,38,39]. There is robust evidence that firefighters are regularly exposed to hazardous noise levels exceeding the exposure limits recommended by the National Institute for Occupational Safety and Health (NIOSH) [40]. Routine hazardous noise exposures in the fire service include sirens, water pumps, saws, alarms, engines and radios, and other equipment or machinery [35]. This is complicated by the underutilization of hearing protection [41] and the prioritization of situational awareness over the perceived future risk of acquiring NIHL [36,42]. Despite the known risk of occupational hazardous noise exposure and NIHL in firefighters, to date there are limited data on their hearing profiles and no studies reporting the characteristics of DPOAEs in this group. It is challenging to collect longitudinal data from the same cohort while controlling for factors that may impact long-term outcomes. Therefore, this investigation examined the effects of years of noise exposure on DPOAE amplitude in firefighters, utilizing years in the fire service as a proxy for noise exposure.

Inner ear structural changes can occur years before becoming clinically evident [2,3,4,5], contributing to a poor awareness of the negative health effects of noise, even in at-risk populations [43]. The present study describes the relationships between self-reported auditory symptoms and hearing profiles in an occupationally noise-exposed population, firefighters, by assessing hearing thresholds, DPOAE amplitudes, and hearing health history. We also explored the associations of DPOAEs with self-reported hearing disability and whether these associations vary by age and years working in the fire service.

## 2. Materials and Method

### 2.1. Design

This study was designed as a prospective population-based cohort study. All study procedures were approved by the University of Miami Institutional Review Board (IRB# 20200222).

### 2.2. Participants

We collected self-reported data from 220 South Florida firefighters from a custom 53-item questionnaire on perceptions of noise exposure, hearing and balance health, and noise safety practices. As part of the survey, the demographic data collected were respondent age, gender, race, ethnicity, marital status, and education. All participants were also asked about hearing and balance health history, cognitive symptoms, exposure to noise, and use of hearing protection. Additional information reported by the participants included their current role as a firefighter, military service, occupational and recreational noise exposure, balance and auditory symptoms following exposure to hazardous noise, and use of a hearing assistive device. We further collected functional hearing outcomes in a subgroup of 176 career firefighters recruited from South Florida fire departments during their 2020 annual health examinations and present the data from this cohort here.

### 2.3. Procedures

Auditory measurements were conducted onsite at a local fire station in Miami-Dade County, Florida, during annual physical assessments of fire service members. All measurements were performed by trained research personnel in an enclosed room, free of interference from external noise. Noise levels were measured intermittently throughout testing to ensure that ambient noise did not exceed 45 A-weighted decibels (dBA), thereby meeting the ANSI S3.1–1999 standard for maximum permissible ambient noise levels for audiometric and DPOAE testing [44,45].

Otoscopy was performed in all participants prior to audiologic assessment. Visual examination of the ear canal and tympanic membrane was conducted to ensure normal anatomy and that external canals were free from occluding debris.

Behavioral hearing thresholds were measured using air-conducted pure-tones under noise-reduction insert headphones (ER-3A insert earphones, Etymotic Research Inc., Elk Grove Village, IL, USA) for both ears using an MA40 portable diagnostic audiometer (MAICO, MN, USA) calibrated to meet the American National Standards Institute standards. Pure-tone air conduction hearing thresholds were obtained for test frequencies of 0.5, 1, 2, 3, 4, 6, and 8 kHz in 5-decibel (dB) steps using a modified Hughson–Westlake procedure [46]. Normal hearing was defined as hearing thresholds of <20 dB HL for all the frequencies tested [47]. Hearing thresholds were not assessed below 15 dB hearing loss at any frequency.

DPOAEs were recorded bilaterally to objectively measure cochlear OHC function. DPOAE amplitude was measured using a Bio-Logic Scout AuDX system (Natus Medical Inc., Mundelein, IL, USA). Responses were elicited with two simultaneously presented primary frequency tones with a fixed primary ratio (f_2_/f_1_) of 1.22, L1 = 65 dB sound pressure level (SPL), L2 = 55 dB SPL, with f_2_ frequencies ranging from 1.5 to 10 kHz. An in-the-ear calibration provided by the Bio-Logic test equipment was utilized prior to each measurement. The probe was coupled to the participant’s ear with a disposable foam ear tip. The DPOAE response was considered present when the DPOAE amplitude was greater than 6 dB above the noise floor [48,49,50]. DPOAE responses are reported in dB SPL. Additionally, the DPOAE level should be above the maximum system distortion of approximately −20 dB SPL. As such, DPOAEs of less than or equal to −20 dB SPL are not considered to be responses from the cochlea [51].

### 2.4. Statistical Methods

For the purpose of this investigation, the self-reported outcomes were limited to the following for analysis: (1) Do you have concerns about your hearing (yes—right ear; yes—left ear; yes—both ears; no)? (2) Do you have ringing or buzzing in the ear? (3) Do you experience changes in hearing following loud noise exposure? (4) Do you experience ringing/buzzing in your ear following loud noise exposure? (5) Do you experience muffled hearing following loud noise exposure? Responses for self-reported symptoms were collected on a five-point scale (never, rarely, sometimes, often, and always). For descriptive analysis of self-reported changes in auditory function and DPOAE findings, responses were converted to yes/no binary variables where often–always was considered ‘yes’ and never–sometimes was considered ‘no’.

Descriptive analyses were based on non-missing responses and conducted for demographic variables and the 5 self-reported outcomes. Correlation analyses were used to analyze associations between self-reported changes in hearing function and DPOAE amplitude. Analyses of variance were conducted to examine the relationships between population factors and hearing function. Statistical significance was defined by *p*-values < 0.05. Pairwise comparisons with Bonferroni corrections for multiple comparisons were used to follow up significant main effects. All analyses were performed using the SPSS^®^ software (version 26.0; New York, NY, USA: IBM Corp^®^).

## 3. Results

Participants ranged in age from 21 to 59 years (mean 38.8 ± 8 years), years of service ranged from 1 to 31 years (mean 12.3 + 7 years), and 95% identified as male. In terms of race, 80% identified as Caucasian, 7% as African American, 0% as Asian, 0% as Pacific Islander, and less than 1% as American Indian or Native Alaskan, with the remainder selecting “other” or “Do not know/Unsure”. Nearly 27% of the total population self-identified as Hispanic, whereas the rest identified as non-Hispanic or other. Ninety-five percent of the service members were career firefighters. Participants were classified into three groups: (1) early career defined as less than 10 years in the fire service, (2) mid-career defined as 10–19 years of service, and (3) late career defined as those with 20 years or more of service.

### 3.1. Functional Assessment Findings

**Figure 1:**
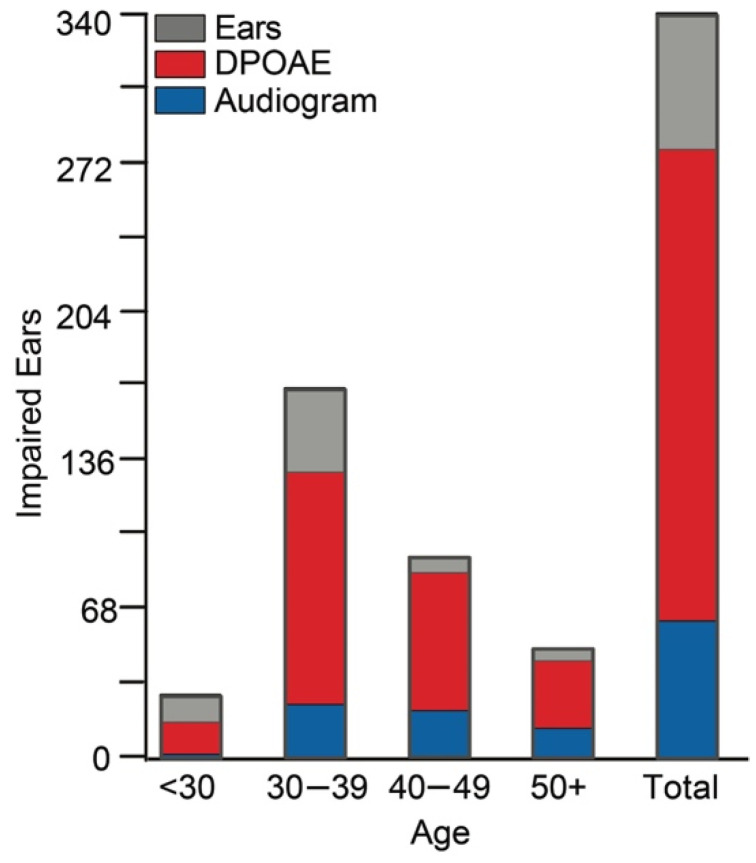
Total count of impaired ears identified out of 340 firefighter ears measured by standard audiogram (blue), as indicated by thresholds >25 dB HL for at least 1 frequency, as well as in DPOAE (red), as indicated by absent responses at 2 or more frequencies, plotted over the total number of ears in each age group (grey). Total impairments for all ears are also presented.

Functional hearing assessment information was obtained for 176 firefighters. Twelve ears could not be assessed due to the presence of occluding cerumen/debris or the visualized perforation of the tympanic membrane, totaling 340 ears for inclusion. Those firefighters with audiometric thresholds exceeding 25 dB for at least one frequency were considered to have impaired hearing. Normal hearing function was indicated in 81% (273/336) of ears in career firefighters by standard audiograms. Conversely, 64% (217/340) of firefighter ears had absent DPOAEs at two or more f_2_ frequencies. Figure 1 presents the proportion of impaired ears, as indicated by thresholds exceeding 25 dB HL at one or more frequencies, compared to the proportion of impaired ears, as indicated by 2 or more absent DPOAE responses. A result was considered absent if the DPOAE was not present with at least 6 dB SNR [48,49,50].

**Figure 2:**
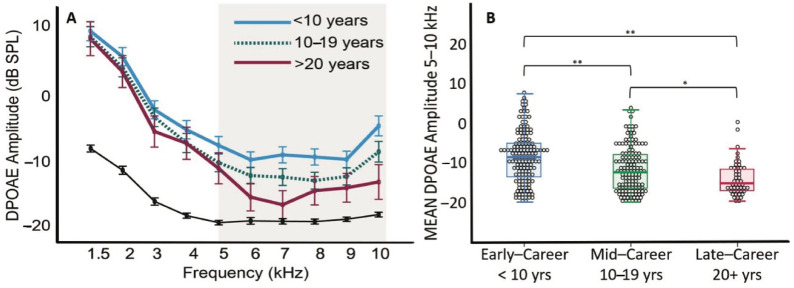
DPOAE results from 340 ears of 176 firefighters. The measurements are reported for early-career firefighters with <10 years of service (blue), mid-career firefighters with 10–19 years of service (green), and late-career firefighters with 20+ years of service (red). In (**A**), DPOAE amplitude by f_2_ frequency is presented (mean ± S.E.M). The grey line represents the average noise floor by frequency. DPOAE amplitude decreases with an increase in frequency and it can be seen that early career firefighters have significantly higher amplitudes than mid- (*p* < 0.001) and late-career firefighters (*p* < 0.001), with significance represented by grey bar at frequencies between 5 and 10 kHz. In (**B**), the mean and inter-quartile range of the average DPOAE amplitude from 5 to 10 kHz are presented by group. Significant differences in mean DPOAE amplitude were observed between the early-career and the mid-career firefighters (**, *p* < 0.001), early-career and the late-career firefighters (**, *p* < 0.001), and the mid-career and late-career firefighters (*, *p* < 0.05).

Analyses revealed the significant effect of years in the fire service (*F*(2, 327) = 12.03, *p* < 0.001, *ηp*^2^ = 0.069), with DPOAE amplitude decreasing with increasing years of service (Figure 2A). As can be seen in Figure 2A, the largest effects for DPOAEs occurred above 5 kHz. Figure 2B presents the mean and inter-quartile range of the average DPOAE amplitude from 5 to 10 kHz by group. Multiple comparisons with Bonferroni adjustment (Figure 2B) revealed a mean difference of 2.9 dB SPL between the early-career and the mid-career firefighters (*p* < 0.001) and a mean difference of 5.4 dB SPL between the early-career and the late-career firefighters (*p* < 0.001). Mean DPOAE amplitude decreased by 2.5 dB SPL in late-career firefighters compared to mid-career firefighters (*p* < 0.05). We also observed that the total number of frequencies where DPOAE responses were absent increased with increasing years in the fire service (*F*(1, 330) = 6.97, *p* < 0.01).

When looking at age as a factor, correlation analysis revealed that age was significantly associated with reduction in DPOAE amplitude for the frequency range of 5–10 kHz, although this only accounted for a small portion of the variance (*r*^2^ = 0.139, *p* < 0.01). While the study population ranged in age from 21 to 59 years, the vast majority (78%) of the population was under 45 years of age. Thus, age was likely not the main factor in the DPOAE findings for this cohort [52,53]. To quantify the results statistically, two-way ANOVA was performed to analyze the effect of age and years of service on DPOAE amplitudes in the frequency range of 5–10 kHz. There was a significant main effect of years of service (*F*(2, 330) = 6.06, *p* < 0.01, *ηp*^2^ = 0.036), but no main effect of age (*F*(3, 329) = 1.39, *p* = 0.25, *ηp*^2^ = 0.013), and no interaction of years of service by age (*F*(3, 329) = 6.06, *p* = 0.05, *ηp*^2^ = 0.024). Furthermore, mean DPOAE amplitude did not vary significantly by age for those with less than 10 years of service. Specifically, firefighters in the highest age brackets (40–49 years and 50+ years) with 10 or more years of service had significantly reduced mean DPOAE amplitudes compared to their age-matched firefighter cohorts with <10 years of service (−14.89 + 1.22 dB SPL; −6.47 + 2.43 dB SPL, *p* < 0.001).

**Figure 3:**
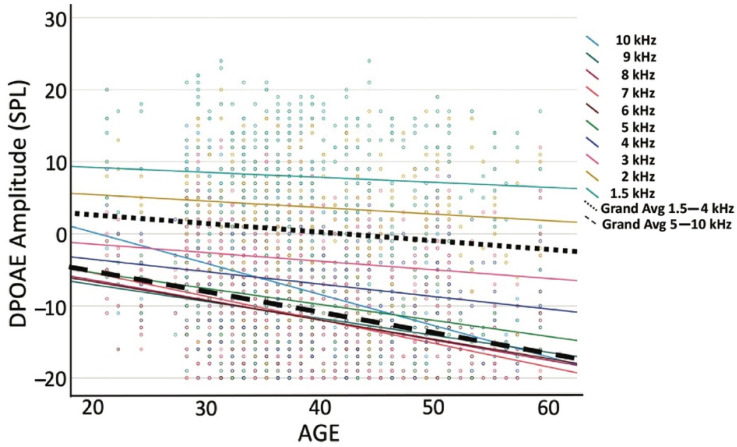
DPOAE amplitudes by age of the firefighters. The scatter plot shows DPOAE amplitude from all firefighters where age was reported, totaling 328 ears colored by frequency (f_2_). The grand averages of DPOAE amplitudes for f_2_ between 1.5 kHz and 4 kHz and f_2_ between 5 kHz and 10 kHz are highlighted with two thick black dashed lines.

Figure 3 shows the frequency-specific absolute DPOAE amplitudes by age. There was little change in the lower frequencies as a function of age. However, a greater DPOAE amplitude reduction was observed among this firefighter group with increasing frequency. The grand averages of mean DPOAE amplitude for the frequency range of 1.5–4 kHz were between 5 dB SPL for firefighters near 20 years of age and −3 dB SPL for those above 60 years. At the higher frequencies, 5–10 kHz, the grand averages ranged from −6 dB SPL for those near 20 years to −17 dB SPL for those above the age of 60 years. Simple linear regression was fit to predict DPOAE amplitude based on age. For low frequencies, the *F*(1, 326) = 5.52, *p* < 0.05, *R*^2^ = 0.017, whereas for the higher frequencies the *F*(1, 326) = 52.50, *p* < 0.001, *R*^2^ = 0.139. Even with a gradual slope, age significantly predicted DPOAE amplitude at both the lower frequency range of 1.4–4 kHz (*β* = −0.113, *t* = −2.35, *p* < 0.05) and the higher frequency range of 5–10 kHz (*β* = −0.294, *t* = −7.25, *p* < 0.001), indicating that for every year increase in age, DPOAE amplitude reduced by −0.113 at the lower frequencies and −0.294 at the higher frequencies.

### 3.2. Reported Outcomes

**Figure 4:**
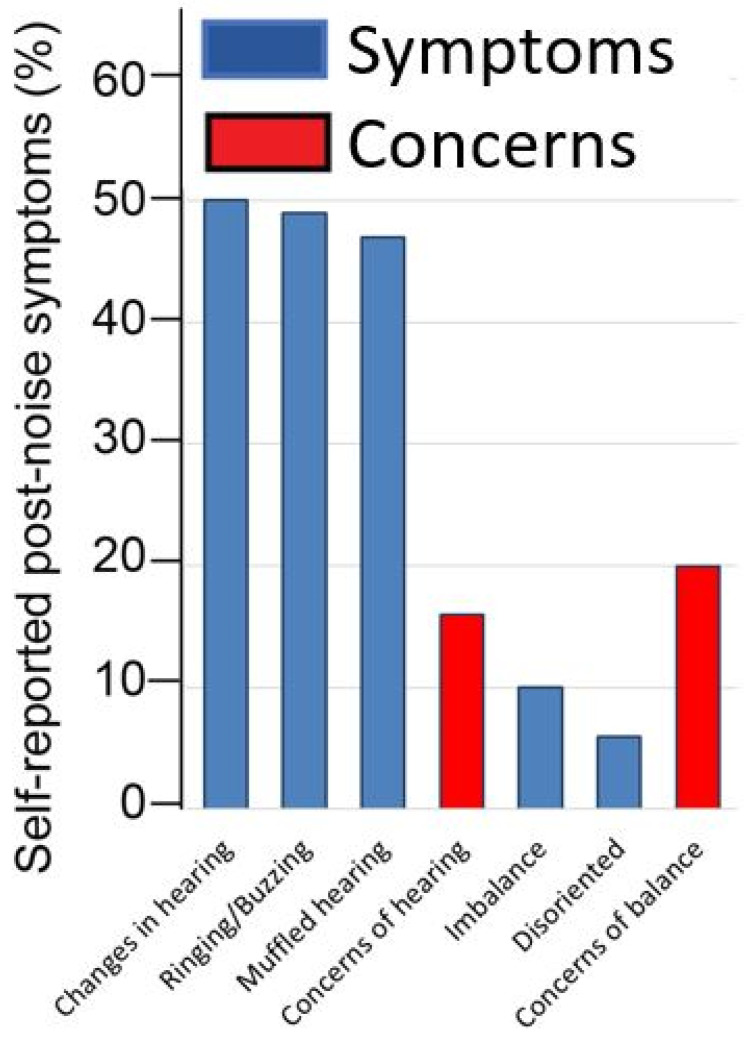
Self-reported changes in hearing and balance following hazardous noise exposure in firefighters are presented in blue and self-reported concerns about hearing and balance are presented in red (*n* = 176).

In total, 6.3% of firefighters who participated in the study reported a prior diagnosis of hearing impairment. Overall, 55% of firefighters reported experiencing some combination of hearing symptoms during their career in the fire service. When assessing hearing symptoms experienced following exposure to hazardous noise (Figure 4), the sensation of muffled hearing following exposure to hazardous noise was reported in 47% of firefighters, 50% reported experiencing perceptual changes in hearing after noise exposure, and 49% reported experiencing ringing after noise exposure. However, only 16% reported concerns about their hearing. When asked about balance, 20% of the firefighters reported concerns about their balance, while 10% reported experiencing episodes of imbalance after noise exposure and 6% reported being disorientated after noise exposure.

Spearman’s rank correlation was performed to explore the potential relationships between self-reported hearing symptoms and DPOAE. No significant correlations were observed for the presence of ringing or buzzing in the or for auditory symptoms experienced after noise exposure with self-reported DPOAE (Table 1). Bonferroni correction was applied, so all effects are reported at a 0.005 level of significance.

### 3.3. Relation between Self-Report Concerns about Hearing Loss and Functional Assessments

To explore the relationship between self-reported concerns about hearing and DPOAE amplitude, responses were then converted to binary variables and an independent-samples *t*-test was conducted to compare DPOAE amplitude in firefighters who had no concerns about their hearing to firefighters who self-reported concerns about hearing loss. Results found that firefighters who had concerns about hearing loss had lower DPOAE amplitudes (i.e., responses) than firefighters who did not have concerns about their hearing (*t* = −2.707, *p* < 0.01, *d* = 0.4). Although imbalance is a less recognized consequence of noise exposure [42], firefighters demonstrated higher rates of concern regarding balance compared to hearing (20% vs. 16%, respectively).

## 4. Discussion

Noise exposure is a recognized modifiable risk factor contributing to the development of age-related hearing loss [2,6,7,8]. In a large cohort of young career firefighters, we found increasing cochlear OHC dysfunction with increased years served in the fire service. DPOAEs provide an objective measure of cellular function at frequency-specific regions of the inner ear. In humans, DPOAEs are used for the early and differential diagnosis of damage to the cochlear OHCs [23,54,55,56]. A significant decrease in DPOAE amplitude with increasing years in the fire service was observed (Figure 2). The largest effects for DPOAEs in our cohort were observed above 4 kHz, as is typical in the general population [57] and other occupational workers. Seixas et al. (2005) showed similar findings in a young cohort (27.5 ± 6.4 years) of early-career occupational workers where DPOAE amplitudes reduced over time while controls remained relatively unchanged and showed significantly higher amplitude responses. When looking at the high-frequency DPOAE data presented in Figure 3, the slope shows amplitude changes starting very early, within the first decade of fire service, whereas the low-frequency responses remain largely unchanged. This can also be observed in Figure 1, where a significant proportion of firefighters present with absent DPOAEs regardless of age group. Collectively, this demonstrates the increased sensitivity of DPOAEs compared to standard hearing tests (i.e., audiograms). The review of the high-frequency DPOAE data suggests there an accelerated aging of ears occurs within this population, highlighting the importance of assessing higher-frequency regions in monitoring at-risk populations for NIHL.

Noise is ubiquitous in the fire service and is a leading cause of acquired NIHL in this group, particularly over the span of a long career [38,41]. The recommended exposure limit for noise is 85 dBA as an 8 h time-weighted average, above which exposures are considered hazardous [40]. Firefighters routinely experience sound levels higher than this limit via sirens and alarm tones, water pumps, saws, emergency response vehicles, and other equipment or machinery that generates excessive noise [35].

There is mounting evidence that such repeated or prolonged exposure to noise can lead to inner ear structural changes in the presence of normal hearing. This “hidden hearing loss” precedes detectable hearing loss [2]. Our data present a high incidence of subclinical hearing deficits in a cohort of otherwise healthy firefighters. These findings are consistent with those reported by others [20,22,58,59], indicating that DPOAEs, in combination with standard audiometric measures, could be used to improve surveillance for NIHL in at-risk occupational groups [58,60,61,62]. Our data provide evidence of preclinical damage occurring very early in career firefighters, indicative of early auditory aging. In Figure 2B, it can be observed that a significant portion of individuals in their early careers present with a small DPOAE amplitude (i.e., <−5 dB SPL), and this becomes more prevalent in the mid- and late-career groups. Indeed, as firefighters progress in their career, they also age, leading to an unavoidable noise–age interaction where years of service (i.e., exposure) is a driving factor.

Noise injury is also associated with temporary shifts in hearing sensitivity. Repeated overexposures lead to a permanent change in hearing over time, making temporary changes in hearing after exposure a reliable indicator of noise-induced damage to the ear [63]. Temporary threshold shifts are characterized by temporary sensations of reduced hearing, muffled hearing, and/or having ringing or buzzing in the ear [18]. Temporary threshold shifts occur with hazardous noise exposure but typically recover in short order, both perceptually by the listener as well as in behavioral measures of hearing sensitivity such as an audiogram within 48 h [8]. Due to this clinical presentation, listeners are often unaware of the potential underlying noise injury or long-term health effects [43]. This was reflected in the self-reported perceptions of firefighters, who, despite reporting high rates of auditory symptomology consistent with incidents of noise injury, were largely not concerned about their hearing. However, firefighters who self-reported concerns about their hearing had reduced DPOAE amplitudes from 5 to 10 kHz compared to firefighters who did not have concerns about their hearing (*p* < 0.01). Perceived changes in hearing sensitivity were not found to be correlated with DPOAE amplitude. However, the lack of relationship may be related to poor recall or the under-recognition of auditory symptoms as negative health consequences, particularly when they are subtle or less overt. Presumed healthy individuals may be more likely to dismiss symptoms or interpret them as insignificant, which in turn may influence their recall. In general, noise is under-recognized as a potential health hazard [43]. It is possible that the predictive value of perceptual changes in hearing sensitivity may improve with increased awareness of the risk associated with hazardous noise exposures or the long-term consequences of NIHL.

In general, firefighters are highly aware of the common symptomology characterizing noise injury, with 55% reporting experiencing at least one auditory symptom following hazardous noise exposure. However, they seemingly do not perceive these symptoms as an actual health hazard, based on the small proportion reporting concerns about their hearing (16%). It is also possible that the determination of “normal hearing” from audiometric findings leads firefighters to under-recognize signs of noise injury. Here, 48% of firefighters reported tinnitus, yet less than 20% had measurable hearing loss on audiometry.

Interestingly, firefighters were more concerned about balance than hearing, despite not recognizing imbalance as a potential consequence of hazardous noise exposure [42]. This may be due to their heightened awareness of the importance of balance for their everyday duties [42] and the high rates of injuries in the fire service subsequent to falls [64]. In fact, studies suggest that imbalance is the most common cause of work-related injury in firefighters [65]. The frequency and severity of injuries also increase with age, a known risk factor for work-related injury in firefighters [66]. These are important considerations in the firefighter population. In this cohort, 10% reported experiencing episodes of imbalance after noise exposure and 6% reported being disorientated after noise exposure, highlighting the assessment and surveillance of balance as potentially important components of total worker health. Future studies should consider the effects of hazardous noise exposure in at-risk groups such as firefighters.

As the aging ear is also subject to changes to the sensory structures of the inner ear, such as OHC loss and atrophy of the stria vascularis [2,48,52,53], these results could be confounded by the inherent difficulty in separating the aging effect from the effect of noise exposure [6]. However, unlike in previous studies [67,68], age was not found to be a reliable predictor of changes to the inner ear. This is likely due to our use of a comparatively young cohort, which removed other potentially contributing factors such as age and illness. The substantial number of firefighters with DPOAE abnormalities in the presence of normal hearing highlights the significant risk occupational groups such as firefighters face for developing NIHL. This is of importance, as standard tools (i.e., audiograms) failed to detect the subclinical markers of hearing loss in most of this population.

Noise exposure is a modifiable risk factor for hearing loss. Evidence shows that the successful management of modifiable risk factors can be effective in both delaying or preventing disease and reducing healthcare costs [18]. Our results highlight that standard audiometric testing is not a reliable means of surveillance with which to detect early noise injury in at-risk occupational groups such as firefighters. As reported by others [69], reliance on self-reported hearing is also not a reliable means of monitoring for changes in hearing status. Previous studies have indicated that measured degree of impairment is not correlated with increased rates of treatment, and that individuals with hearing loss may not appropriately self-identify [69]. Low awareness of the adverse health effects of noise, combined with the delayed and heterogenous presentation of clinical symptoms, is a critical barrier.

It is well established that hazardous noise exposure leads to an acceleration of age-related hearing loss and that noise injury leads to the inner ear being more susceptible to early aging [2,70,71]. This is of particular concern, as a growing body of evidence points to hearing loss as a risk factor for cognitive decline [72]. Risk of neurocognitive disorder increases by three times with mild hearing loss and five times with severe hearing loss [15]. Of the 12 modifiable risk factors, hearing loss presents the greatest opportunity to reduce dementia prevalence, more so than depression, social isolation, hypertension, and others [72]. Given the prevalence of age-related hearing loss and the established links to early exposure to hazardous noise, early monitoring and prevention of exposures presents an important area of focus for improving the long-term health of occupational workers and leading to healthy aging.

A primary limitation of our study is the lack of an age-matched control group. There are also no reliable comparison data available for accumulated noise exposure over the years. We also acknowledge the potential effects of ototoxic agents on hearing loss in the fire service. However, we note that our focus was on examining the effects of years of service, which serves as a proxy for total noise exposure, on absolute DPOAE amplitude. We also note the inherent challenges of conducting a field study in a large cohort of occupational workers where time constraints prevent the implementation of more comprehensive test batteries. While we controlled the environmental factors that can adversely impact the measurements, challenges remain in implementing these sensitive measures as surveillance tools in the field with potential impact on test–retest reliability.

## 5. Conclusions

- The characterization of DPOAE in a group of relatively young firefighters suggests that firefighters are at risk of NIHL due to their repeated exposure to hazardous noise during their service.
- Findings indicate that noise injury occurs early and is not detected by standard surveillance methods.
- The significant associations between subclinical hearing deficits and number of years in service highlight the importance of early detection measures and the need to address the role of a critical modifiable risk factor, noise overexposure, in mitigating hearing loss and reducing the prevalence of cognitive impairment in aging adults.

## Figures and Tables

**Table 1:** Descriptive statistics and correlation coefficients for self-reported hearing symptoms and average DPOAE amplitude from 5 to 10 kHz.

Variable	Mean	SD	1	2	3	4
1. Ringing/buzzing ITE	1.71	0.965				
2. Muffled hearing AEHN	1.65	0.830	0.419 *			
3. Changes in hearing AEHN	1.69	0.823	0.211 *	0.31 *		
4. Ringing or buzzing ITE AEHN	1.74	0.868	0.284 *	0.334 *	0.784 *	
5. DPOAE amplitude 5–10 kHz	−10.71	6.176	−0.095	−0.057	−0.085	0.001

* *p* < 0.001 (2-tailed); ITE—in the ear; AEHN—after exposure to hazardous noise.

## Data Availability

Derived data supporting the findings of this study are available from the corresponding author (HS) upon request.
